# Structural basis of a novel PD-L1 nanobody for immune checkpoint blockade

**DOI:** 10.1038/celldisc.2017.4

**Published:** 2017-03-07

**Authors:** Fei Zhang, Hudie Wei, Xiaoxiao Wang, Yu Bai, Pilin Wang, Jiawei Wu, Xiaoyong Jiang, Yugang Wang, Haiyan Cai, Ting Xu, Aiwu Zhou

**Affiliations:** 1 Hongqiao International Institute of Medicine, Shanghai Tongren Hospital/Faculty of Basic Medicine, Key Laboratory of Cell Differentiation and Apoptosis of The Chinese Ministry of Education, Shanghai Jiao Tong University School of Medicine, Shanghai, China; 2 Alphamab Co. Ltd., Suzhou, Jiangsu, China

**Keywords:** Immunotherapy, Nanobody, PD-L1, Structural Biology

## Abstract

The use of antibodies to target immune checkpoints, particularly PD-1/PD-L1, has made a profound impact in the field of cancer immunotherapy. Here, we identified KN035, an anti-PD-L1 nanobody that can strongly induce T-cell responses and inhibit tumor growth. The crystal structures of KN035 complexed with PD-L1 and free PD-L1, solved here at 1.7 and 2.7 Å resolution, respectively, show that KN035 competes with PD-1 (programmed death protein 1) for the same flat surface on PD-L1, mainly through a single surface loop of 21 amino acids. This loop forms two short helices and develops key hydrophobic and ionic interactions with PD-L1 residues, such as Ile54, Tyr56 and Arg113, which are also involved in PD-1 binding. The detailed mutagenesis study identified the hotspot residues of the PD-L1 surface and provides an explanation for the stronger (~1 000-fold) binding of KN035 to PD-L1 than PD-1 and its lack of binding to PD-L2. Overall, this study reveals how a single immunoglobulin-variable scaffold of KN035 or PD-1 can bind to a flat protein surface through either a single surface loop or beta-sheet strands; and provides a basis for designing new immune checkpoint blockers and generating bi-specific antibodies for combination therapy.

## Introduction

Immune checkpoints are inhibitory pathways of the immune system that maintain self-tolerance and prevent autoimmunity [1–5]. The PD-1/PD-L1 immune checkpoint, which is one of the best characterized receptor ligands in immuno-oncology, involves PD-1 (programmed death protein 1, also known as CD279) [6, 7] and two known ligands PD-L1 (B7-H1; CD274) [8, 9] and PD-L2 (B7-DC; CD273) [10, 11]. PD-1, a type 1 transmembrane receptor, is expressed on the surface of activated T cells in peripheral tissues. Its ligands, PD-L1 and PD-L2, are commonly expressed on the surface of dendritic cells or macrophages [8, 12, 13]. The binding of PD-1 to its PD-L1 ligand limits T-cell activity, thereby preventing excessive stimulation and maintaining immune tolerance to self-antigens [12, 14, 15].

However, these immune checkpoints are often upregulated by tumor cells to induce local immune suppression and attenuate the endogenous antitumor immune response [16, 17]. For example, PD-L1 is often overexpressed in different tumors, including melanoma, lung and breast cancer, which leads to an immune response handicap within the tumor microenvironment [12, 18, 19]. The PD-1/PD-L1 interaction inhibits T-lymphocyte proliferation, cytokine release and cytotoxicity, resulting in the exhaustion and apoptosis of tumor-specific T cells [19]. However, blocking the PD-1/PD-L1 interaction results in the reversal of the exhausted T-cell phenotype and the normalization of the antitumor response [18, 20, 21]. The PD-1/PD-L2 interaction is thought to be essential in maintaining peripheral tolerance, particularly in the lung. PD-L2^−/−^ mice do not express PD-L2, leading to increased airway hyperactivity and lung inflammation [22]. Therefore, specifically targeting PD-L1 without affecting the normal function of PD-L2 may be more advantageous than completely blocking the interactions of PD-1. Recently, immunotherapy using antibodies that block the PD-1/PD-L1 pathway have shown impressive clinical outcomes with durable tumor regression and improved patient survival [1–3,  21], leading to several PD-1 antibodies (such as nivolumab and pembrolizumab) and PD-L1 antibodies (such as atezolizumab, durvalumab and BMS-936559) in clinical use or in late-stage clinical development [23]. Here, we have screened and identified a specific high-affinity PD-L1 nanobody (single domain antibody) [24], designated KN035, which shows strong antitumor activity comparable to that of durvalumab, a fully humanized antibody developed by AstraZeneca showing promising readouts in multiple phase III clinical trials [25, 26]. Notably, the smaller size and favorable physico-chemical properties of KN035 offer great potential in many immuno-oncology applications. Furthermore, the crystal structure of this nanobody complexed with human PD-L1, solved here, reveals the binding mechanisms between KN035 and PD-L1, and explains their high binding affinity and specificity, which provides a basis for the *de novo* design of PD-1/PD-L1 inhibitors.

## Results

### Screening and identification of human PD-L1 nanobody KN035

**Figure 1:**
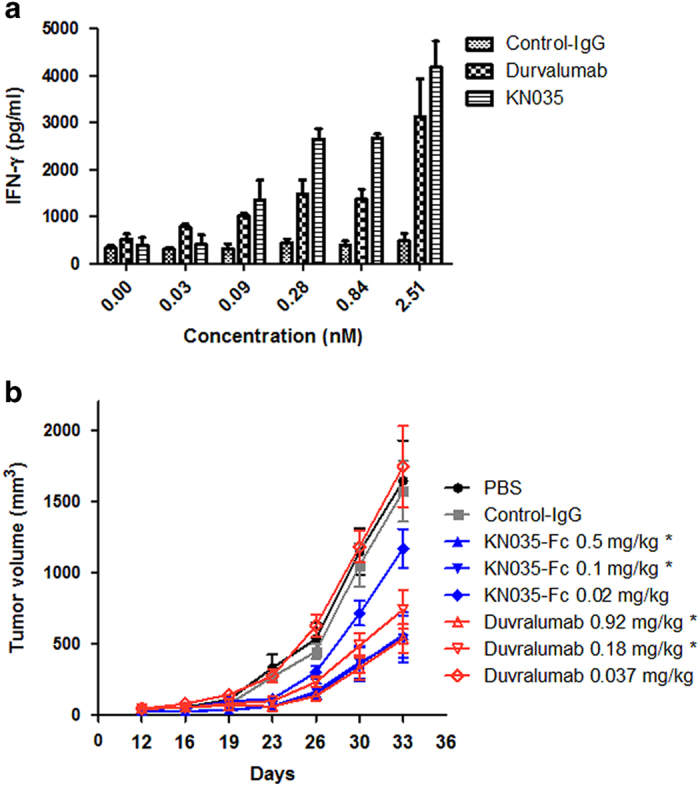
Activity of KN035-Fc assessed by T-cell response and tumor growth inhibition. (**a**) The levels of IFN-γ secreted by CD4+ T cells following treatment with different concentrations of KN035-Fc, durvalumab or control IgG were measured by ELISA and plotted against the antibody concentrations. Although KN035-Fc appears to have higher activities than durvalumab at 0.84 and 2.51 nM, the differences were not statistically significant, with *P-*values of 0.051 and 0.054, respectively. The data are shown as the means ±s.e.m. (*n*=3). (**b**) The therapeutic effect of different doses (same molar amount) of KN035-Fc or durvalumab was assessed in xenograft tumor models where a mixture of A375-PD-L1 cells and PBMC at a ratio of 4:1 (total 5×10^6^ cells), was inoculated into mice with KN035-Fc or durvalumab, intraperitoneally four times over 2 weeks. Tumor growth was measured every 3 or 4 days. KN035-Fc showed a weak antitumor effect at the concentration of 0.02 mg kg^−1^ and a strong antitumor effect at high doses (0.1 mg kg^−1^ and 0.5 mg kg^−1^), whereas durvalumab showed antitumor activity at high concentrations (0.18 mg kg^−1^ and 0.92 mg kg^−1^). Control IgG antibody had no antitumor activity. *P<0.05 compared with the PBS group. The data are shown as the means ±s.e.m. (*n*=8).

To obtain high-affinity PD-L1 antibody, we immunized camels with human PD-L1 (termed PD-L1 if not specified) and screened the heavy-chain-only nanobody phage library derived from a PBMC (peripheral blood mononuclear cell) with conventional panning technology [27]. One of the identified binding nanobodies, named KN035, has high thermal stability with a thermal melting point of 66 °C (Supplementary Figure S1A). Its fusion protein with the Fc fragment of human IgG1, KN035-Fc, binds PD-L1 specifically, with a *K*d value of 3.0 nM (Supplementary Table S1, Supplementary Figure S1B). KN035-Fc binds specifically to human PD-L1, and KN035 shows no cross reactivity with other members of the same family or with the mouse PD-L1 when assessed by flow cytometry (Supplementary Figure S2). KN035-Fc blocks the interaction between PD-L1 and PD-1 with an IC_50_ value of 5.25 nM in a competitive ELISA. It competitively displaced human PD-1 (termed PD-1 if not specified) from pre-formed PD-1/PD-L1 complexes slightly more effectively than durvalumab (Supplementary Figure S3), which is likely due to its smaller size. Similar to durvalumab, KN035-Fc could dose- and time-dependently induce T-cell cytokine production in a mixed lymphocyte reaction *in vitro* (Figure 1a and Supplementary Figure S4). As KN035-Fc is specific to PD-L1 and does not bind mouse PD-L1, its *in vivo* activity could not be directly analyzed in mice. Therefore, an immune xenograft tumor model previously used for durvalumab characterization [28] was chosen for assessing the *in vivo* activity of KN035-Fc. Tumor A375 cells overexpressing PD-L1 were mixed with human PBMCs and were used to subcutaneously inoculate NOD-SCID mice. As shown here, durvalumab, when intraperitoneally administered, inhibits tumor growth at 0.18–0.92 mg kg^−1 ^dosages (Figure 1b), which is consistent with a previous report [28]. Similarly, KN035-Fc, with a half-life of ~72 h *in vivo*, also shows strong, if not better, antitumor activity at comparable dosages (0.1–0.5 mg kg^−1^, which is the same molar amount of antibody as durvalumab) (Figure 1b). These results suggest that KN035-Fc is a potent inhibitor of the PD-1/PD-L1 interaction with strong antitumor activity.

### Overall structure of the KN035/PD-L1 complex

**Figure 2:**
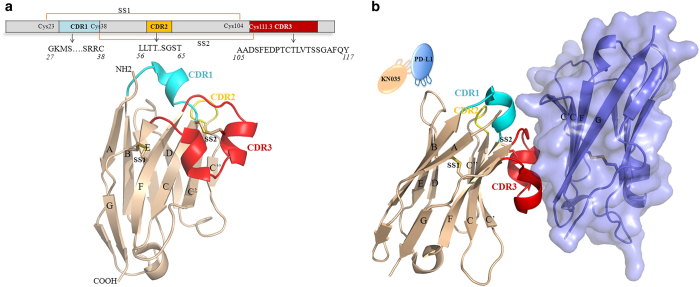
Overall structure of KN035/PD-L1. (**a**) Sequence and structure of KN035. The locations of the CDR1, CDR2 and CDR3 variable regions of KN035 and the positions of disulfide bridges (SS1 and SS2) are indicated. The backbone of KN035 is colored in wheat, and its CDR1, CDR2 and CDR3 regions are colored in cyan, yellow and red, respectively. (**b**) Structure of the KN035/PD-L1 complex. PD-L1 is shown as a slate, semi-transparent surface. The secondary structures of PD-L1 and KN035 are numbered as previously described [31, 34].

To further investigate the molecular mechanism underlying the PD-L1/KN035 interaction, we solved crystal structures of KN035 complexed with an (IgV) *N*-terminal immunoglobulin-variable domain of PD-L1 at 1.7 Å resolution and with free PD-L1 at 2.7 Å resolution. The models were built and refined to obtain good geometry (Table 1). The crystal structure of the KN035/PD-L1 complex contains KN035 and the IgV domain of PD-L1 with 1:1 stoichiometry in the asymmetric unit. Similar to other nanobodies, KN035 shares the typical IgV scaffold containing four framework regions that form the core structure of the immunoglobulin domain [24] and three hypervariable CDR1, CDR2 and CDR3 loops of 8, 8 and 21 residues, respectively [29, 30] (Figure 2a and Supplementary Figure S5). The overall structure of KN035 superimposes well with previously published nanobody structures (PDB: 1MEL, 1HCV) [31, 32] and has root-mean-square deviations ranging between 0.72 and 0.92 Å for the Cα atoms of all residues excluding those of CDR loops. Similar to other nanobodies from camelids, KN035 has a conserved disulfide bond connecting strands B and F (SS1: Cys23-–Cys104). The CDR1 loop of KN035 forms a short alpha helix, whereas the CDR3 loop uniquely adopts a short alpha helix and a short 3_10_ helix, which is not observed in other nanobodies. The short alpha helix of the CDR3 loop is connected to the CDR1 of KN035 by an additional disulfide bond (SS2: Cys38–Cys111.3) (Figure 2), and the CDR3 loop is further stabilized through its hydrophobic interactions with the body of KN035.

**Figure 3:**
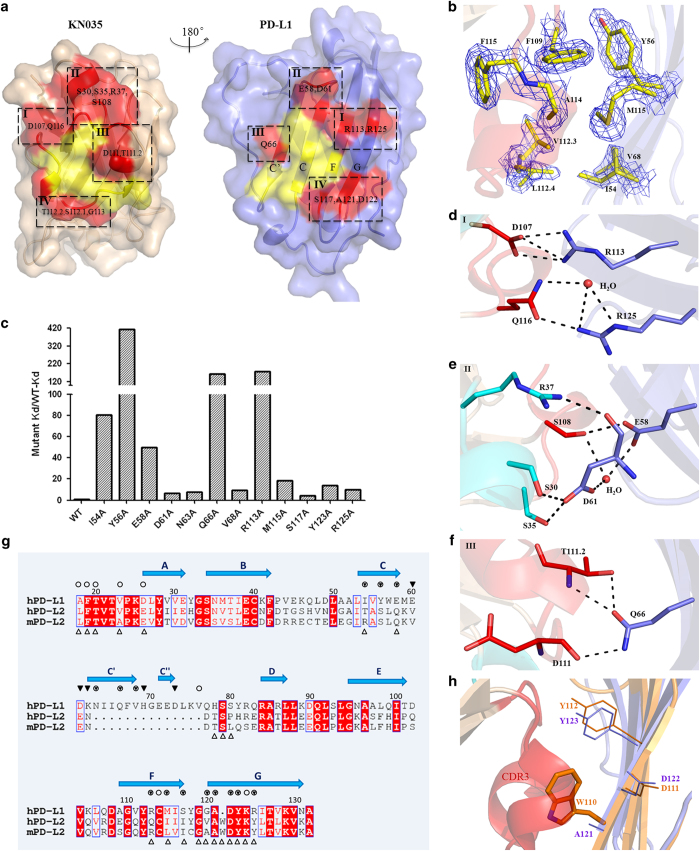
The binding interface of the KN035/PD-L1 complex. (**a**) Open view of the binding surfaces of KN035 (left) and PD-L1 (right) showing the complementary hydrophobic patches (yellow, **b**) and hydrophilic regions (red). (**b**) The clear electron density map shows the phenol ring of F109 in KN035 stacked with the aromatic ring of Y56 in PD-L1 and F115 of KN035, which are further stabilized by neighboring hydrophobic residues A114 and L112.4 from KN035 and M115, I54 and V68 of PD-L1. Detailed interactions from the corresponding regions in **a** (I, II and III) of the hydrophilic surface are shown in (**d**), (**e**) and (**f**) and the IV section is not shown for simplicity. All PD-L1 residues involved in KN035 binding were mutated to Ala individually and were assessed for their contribution to KN035 binding. The changes in the Kd values are plotted in (**c**). (**g**) The structure-based sequence alignment of PD-L1 and PD-L2 is shown; PD-L1 residues that bind PD-1 are highlighted with open circles, and residues interacting with KN035 are highlighted with filled triangles. The overlapping residues are highlighted with triangles in circles. The PD-L2 residues that bind PD-1 are highlighted with open triangles. (**h**) The overlaid structures of PD-L1/KN035 (PD-L1 in blue) with PD-L2 (orange, PDB 3BOV) indicate potential clashes between W110 of PD-L2 and the CDR3 loop (in red) of KN035, which would prevent KN035 from binding PD-L2.

KN035 binds to the IgV domain of PD-L1 with its CDR1 and CDR3 packing against the surface formed by the CC’FG strands of PD-L1 (Figure 2b), with the burial of a total surface area of 1.245 Å^2^ (Figure 3a). The binding of KN035 induces minor main chain conformational changes in PD-L1 when compared with the free PD-L1 structure solved here or to previous structures of PD-L1 (Supplementary Figure S6) [33, 34]. The loop linking strand C and C’ of PD-L1 bends ~2 Å to form interactions with KN035 in the KN035/PD-L1 complex, and there are minor movements of the PD-L1 sidechains during KN035 binding. The connecting loop linking strands C’ and D of PD-L1 has shifted ~7.5 Å, which is likely caused by crystal packing (Supplementary Figure S6). Overall, these findings indicate that the binding surface of PD-L1 is relatively rigid.

### KN035/PD-L1 interaction surface

The CDR1 and CDR3 loops of KN035 form a binding surface with a hydrophobic patch surrounded by hydrophilic surfaces, which is complementary to that of PD-L1 (Figure 3a and Supplementary Figure S7A). A pronounced π–π stacking interaction is observed in which the Phe109 aromatic ring of KN035 is perpendicularly stacked with that of Tyr56 of PD-L1 (Figure 3b), which is further stabilized by other hydrophobic residues (Leu112.4, Val112.3, Ala114 and Phe115) in CDR3 of KN035 and Ile54, Val68 and Met115 of PD-L1. Mutagenesis studies and subsequent affinity measurement experiments (Figure 3c) show that the replacement of Tyr56 by Ala in PD-L1 reduces its binding affinity towards KN035 by >400-fold and that mutation of Ile54 to Ala in PD-L1 reduces the binding affinity by 80-fold (Figure 3c and Supplementary Table S1). Furthermore, KN035 forms close to seven hydrogen bonds and two ionic bonds with PD-L1, involving nine KN035 residues and six PD-L1 residues (Supplementary Table S2). These polar interactions include strong salt bridges between Asp107 of KN035 and Arg113 of PD-L1, with the sidechains of both residues fully extended and stabilized by surrounding residues (Figure 3d). Replacement of Arg113 with an Ala reduces the binding affinity of KN035 to PD-L1 by nearly 178-fold. The importance of this salt bridge of Arg113 for KN035 binding is emphasized by the observation that KN035 has a negligible binding affinity towards mouse PD-L1, which has a Cys at position 113 (Supplementary Figure S2). Glu58 of PD-L1 forms two hydrogen bonds with Ser108 of KN035 (Figure 3e), and Gln66 in the C’ strand of PD-L1 forms three hydrogen bonds with the main chain or sidechain of Thr111.2 and Asp111 of KN035 (Figure 3f). Similar replacement of Glu58 and Gln66 with Ala in PD-L1 decreases the KN035 binding affinity by 50- and 162-fold, respectively. Therefore, these five residues (Figure 3c) likely represent the so-called hotspot residues of the PD-1/PD-L1 binding interface [35, 36]. Furthermore, the sidechains of these residues in the KN035/PD-L1 complex largely adopt similar conformations as observed in the free form of PD-L1 structures solved here or previously published (Supplementary Figure S6). The limited sidechain conformational change during complex formation likely consumes little free energy and favors tight binding. Crystal structures of the hPD-Ll/Avelumab and PD-L1/BMS-936559 show that these antibodies bind PD-L1 through 5-CDR loops [37, 38] instead of two CDRs in KN035. Although there is no detailed characterization of the binding interface, the binding sites of these antibodies cover PD-L1 residues identified here, such as Glu58, Tyr56, Gln66 and Arg113. This finding further supports that these residues are hotspot residues of the PD-L1 surface.

Other residues of PD-L1 involved in forming hydrophobic or polar interactions are also important for stabilizing the KN035/PD-L1 complex because their single mutations resulted in 4- to 18-fold decreases in the binding affinity (Supplementary Table S1). Interestingly, although Asp61 in the connecting loop between strand C and C’ of PD-L1 moves ~2 Å towards KN035 and forms hydrogen bonds with Ser30 and Ser35 in the helix of the CDR1 loop, substitution of this residue with an Ala (Asp61Ala) reduces the binding affinity by merely sixfold (Figure 3c and Supplementary Table S1). This result indicates that the high binding affinity of KN035 towards PD-L1 is predominantly owing to the interactions formed by the CDR3 loop of KN035, with a minor contribution from its CDR1 loop.

Our initial screening has revealed that KN035 binds PD-L1 with nanomolar affinity but does not bind hPD-L2. Based on the structures of the PD-1/PD-L1 and mPD-1/mPD-L2 complexes [33, 34, 39] and the structure of the KN035/PD-L1 complex shown here, the sequences of PD-L1, PD-L2 and mPD-L2 are aligned, with the residues involved in binding highlighted as shown in Figure 3g. PD-L2 has a shorter connecting loop between strands C and D in its IgV domain, and this connecting loop that forms strand C and C’ in PD-L1 is part of the binding surface for KN035. The absence of this loop is expected to decrease the binding of PD-L2 to KN035. More importantly, when the structure of PD-L2 is superimposed with that of the PD-L1/KN035 complex, it becomes apparent that Trp110, which is an important residue in the PD-1/PD-L2 binding interface [39] and is in a similar position to that of Ala121 in PD-L1, would clash with the CDR3 loop of KN035 and prevent PD-L2 from binding owing to its bulky sidechain (Figure 3h). This result indicates that KN035 is a highly specific antibody toward PD-L1 and would have fewer off-target effects *in vivo*.

### Comparison with PD-1/PD-L1 structures

**Figure 4:**
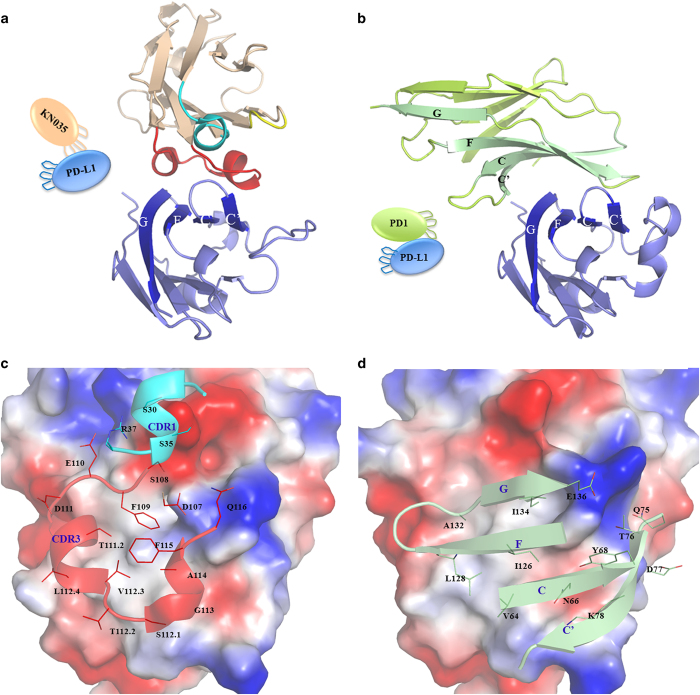
A comparison of PD-L1 binding interfaces with KN035 (**a**, **c**) and PD-1 (**b**, **d**). The IgV domain of KN035 binds PD-L1 through its CDR1 and CDR3 (**a**), and the IgV domain of PD-1 binds PD-L1 through strands C, C’, G and F (**b**). The CDR1, CDR2 and CDR3 of KN035 are highlighted in cyan, yellow and red, respectively. The front and back strands of PD-1 are shown in green and lemon, respectively. The residues of KN035 involved in binding PD-L1 are labeled in (**c**), and the residues of PD-1 involved in binding are shown in (**d**). The electrostatic surface potential of the PD-L1 IgV domain is shown in (**c**) and (**d**) with the negative charges on the surface rendered in red and the positive charges rendered in blue. The PD-1/PD-L1 structure is based on PDB 4ZQK.

It has been shown in previous structures [33, 34, 39] that PD-1, adopting an IgV-type topology, binds PD-L1 through its residues from GFCC' strands (Figure 4a and b and Supplementary Figure S7), with a total buried surface area of 1 500 Å^2^. However, PD-1 binds PD-L1 or PD-L1-Fc relatively weakly, with a *K*d value of 6–8 μM [39, 40], and forms similar interactions with the hotspot residues of PD-L1 as does KN035; its binding interface is largely coincident with that of KN035 (Figure 4c and d). Arg113 in PD-L1 forms a salt bridge with Glu136 of PD-1, which is reminiscent of its salt bridge with Asp107 of KN035 (Figure 3d and Supplementary Figure S7B). However, this salt bridge in the PD-1/PD-L1 complex is relatively weak, and the sidechains of Arg113 and Glu136 are poorly aligned (Supplementary Figure S7B). According to previous mutagenesis studies derived from mouse PD-1 and mouse PD-L1 [39, 41], the ionic interactions from this residue are dispensable in the mPD-1/mPD-L1 interface, and the binding affinity of the corresponding mutant (Cys113Tyr) increased threefold. Similarly, Glu58 has a key role in the binding of PD-L1 to KN035, and the binding affinity of the Glu58Ala mutant decreased 50-fold. However, it is redundant or undesirable for mPD-1 to bind where the Glu58Ser mPD-L1 mutant binds mPD-1 approximately threefold tighter. Furthermore, the hydrophobic interactions between mPD-1 and mPD-L1 appear to be centered on residue 115 (Met115 in humans and Ile115 in mouse) rather than Tyr56 where the Ile115Ala mutant binds mPD-1 ~33-fold weaker than wild-type PD-L1 and the Tyr56Ser mutant binds mPD-1 with the same binding affinity as wild type. By contrast, the key hydrophobic interaction of the KN035/PD-L1 interface is from Tyr56, where similar PD-L1 variants Met115Ala and Tyr56Ala bind KN035 with an affinity decreased by 18-fold and 413-fold, respectively. Furthermore, it has been shown that the hydrophobic interactions between mPD-1 and mPD-L1 could be enhanced by the Ala132Leu substitution in PD-1, leading to an increased binding affinity to both mPD-L1 and mPD-L2 [39]. Therefore, this result may suggest that the binding surface of PD-1 is not optimized or selected by nature to bind PD-L1 with a high binding affinity.

## Discussion

It is now clear that tumor cells often co-opt immune checkpoint pathways as a major mechanism of immune evasion [1–3, 21, 42], particularly against T cells that are specific for tumor antigens. Because the ligand–receptor interactions of these checkpoints could be blocked by antibodies or recombinant ligands or receptors, several antibodies against CTLA-4 and PD-1 have been approved by the FDA for cancer immunotherapy, and many other antibodies are in development [1, 3, 21, 26]. Here, we report the co-crystal structure of such an antitumor PD-L1 nanobody, KN035, in complex with human PD-L1, which paves the way for further antibody optimization for higher binding affinity and specificity.

It was noted from previous structural studies of PD-1 and its ligands that the receptor/ligand binding interface is relatively flat (Supplementary Figure S7B). We have found that KN035 binds the flat surface of PD-L1 mainly through its CDR3 loop, which forms one turn of an alpha helix and a unique short 3_10_ helix. Its nanomolar binding affinity to PD-L1 is mainly achieved through harnessing both hydrophobic and ionic interactions on its binding surface and by making full use of all the residues from the binding interface. For example, residues Asp107 and Phe109 of KN035 are optimally aligned to interact with the corresponding residues Arg113 and Tyr56 from the complimentary binding surface of PD-L1. By contrast, the contribution of these two residues of PD-L1 for PD-1 binding appears minimal (Supplementary Figure S7B and Table S2). Another contributing factor to the large difference in the binding affinities of KN035 and PD-1 to PD-L1 likely arises from the flexibility of the CDR loops, which can interact with residues around the interface, whereas the binding surface of PD-1, as confirmed by previous NMR and crystallographic studies, is mainly centered on GFCC’ beta-strands of relatively limited freedom in main chain conformation [40]. An implication from this finding is that the interface between PD-1 and PD-L1 is not purposefully optimized for maximal binding affinity *in vivo* and that the modest binding affinity of PD-1 to its ligands, in the micromolar range, is derived from a natural selection for transient immune regulation. The same theme could also be applied to other key interactions involved in immune regulation, such as CD28/B7 [43] and T-cell receptor/major histocompatibility complex [44].

Furthermore, the structure of the KN035 and PD-L1 complex explains the lack of binding of KN035 to PD-L2 owing to the shorter PD-L2 loop between strand C and D and the steric hindrance of Trp110 in PD-L2 (Figure 3g and h). Therefore, this specific PD-L1 nanobody could potentially be used for dissecting the roles of PD-L1 and PD-L2 in tumors, which would be crucial in guiding the clinical use of different checkpoint blockers. In addition, a specific PD-L1 antibody will likely have fewer side-effects in clinical use than a direct anti-PD-1 antibody, as it would not affect the normal function of other PD-1 ligands such as PD-L2 [22].

Although various crystal structures of PD-1 complexed with its ligands have been published, the rational design of peptide/small molecule inhibitors to the PD-1/PD-L1 surface have achieved limited success [45], largely owing to the difficulty in targeting the flat surface of a protein–protein interface. Nevertheless, some progress has recently been made with the identification of the binding site of a Bristol-Myers Squibb compound on PD-L1 [45]. The identification of the KN035 binding surface here now provides insight for the further selection of peptides or chemical mimetic based on the configuration of the CDR3 loop. The semi-independent folding of the CDR3 loop may also be utilized to generate bi-specific and multi-specific biologics for combined cancer immunotherapy, which is a major part of next-wave immuno-oncology development [46].

In summary, the data presented here show how the interplay of three IgV domains of KN035, PD-1 and PD-L1 could regulate immune suppression and provide the structural basis for the design and optimization of future immune modulators.

## Materials and Methods

### Generation of camel nanobodies against PD-L1

An anti-PD-L1 nanobody was produced and purified as previously described [27]. In brief, injections of a PD-L1 Fc fusion protein were performed in *Camelus bactrianus*. Peripheral blood lymphocytes (100 ml) were isolated 1 week after the last immunization. The sequence corresponding to the variable domains of the heavy-chain antibodies was amplified with specific primers and used to create a nanobody phage display library. Enrichment screening against PD-L1-Fc was performed in 96-well plates coated with 10 μg of protein per well. High-affinity bacteriophages were obtained after 4 rounds of screening. Ninety-six individual colonies were randomly selected and amplified in culture. Positive colonies verified by ELISA (enzyme-linked immunosorbent assay) were sequenced and defined. The nanobody coding genes were cloned into pET-32b (EMD Biosciences, Shanghai, China) and expressed in *Escherichia coli*. The nanobodies were purified and screened for their ability to block the interaction between PD-L1 and PD-1 by performing a competitive ELISA. One of the nanobodies showing strong activity in blocking the PD-L1/PD-1 interaction was termed KN035. The coding DNA sequence for KN035 was fused with that of the coding fragment for human IgG1 Fc (KN035-Fc) and cloned into vector pcDNA3.1. KN035-Fc was expressed in HEK293 cells and secreted in culture medium. It was purified to homogeneity (>95%) by performing Protein A affinity chromatography (GE Healthcare, Shanghai, China).

### Analysis of IFN-γ production

PBMCs were obtained from heparinized peripheral blood samples of healthy donors using Ficoll–Hypaque density gradient centrifugation [47]. Freshly prepared PBMCs were plated in serum-free Roswell Park Memorial Institute-1640 medium, and incubated at 37 °C in 5% CO_2_ atmosphere for 2–3 h. After incubation, all the suspension cells were carefully removed, and the medium was changed to complete Roswell Park Memorial Institute-1640 medium with 5 ng ml^−1^ granulocyte-macrophage colony-stimulating factor (R&D Systems, Minneapolis, MN, USA) and 20 ng ml^−1^ interleukin-4. The medium was refreshed every 2 days, and 10 ng ml^−1^ tumor necrosis factor-α was added on the 4th day. Finally, the mature dendritic cells were harvested for mixed lymphocyte culture reaction experiments on the 5th day. CD4^+^ T cells (1×10^5^) and allogeneic dendritic cells (1×10^4^) were co-cultured with or without dose titrations of KN035-Fc or the control antibody or durvalumab [28, 48]. The supernatant was collected at various incubation intervals and the levels of IFN-γ were evaluated using an ELISA kit according to the manufacturer’s instructions. The experiments were repeated three times. Means were compared by performing Student’s *t*-tests.

### *In vivo* studies

To evaluate the antitumor effect of KN035-Fc *in vivo*, a xenograft mouse model [28, 47] was prepared by subcutaneously inoculating NOD-SCID mice (6–8 weeks old, eight mice in each group) with a mixture of 4×10^6^ A375-PD-L1 cells (50 μl) and 1×10^6^ human PBMCs (50 μl) [47]. KN035-Fc or durvalumab or control imunoglobulin G or phosphate-buffered saline was intraperitoneally administered at 1, 4, 8 and 12 days after tumor cell inoculation. The doses of KN035-Fc and durvalumab for this experiment were 0.02, 0.1 or 0.5 mg kg^−1^ and 0.037, 0.18 or 0.92 mg kg^−1^, respectively (same molar amount for both). Tumor volumes were measured along three orthogonal axes (a, b and c) and calculated as tumor volume=(abc)/2. Mice with tumor volumes larger than 2 000 mm^3^ were killed by CO_2_ administration. Statistical analyses were performed using GraphPad Prism software (San Diego, CA, USA). The *in vivo* activity of KN035-Fc and durvalumab were also tested on a B16-PD-L1/C57 mouse model; however, both antibodies showed limited tumor-suppressing effect at the same dosages.

### Expression and purification of PD-L1 and its complex with KN035

Genes encoding human PD-L1 (amino acids 19–239) were cloned into pET-28a. Proteins were expressed with a C-terminal His-tag in *E. coli* BL21 (DE3) as inclusion bodies. Cells were cultured at 37 °C in Lysogeny broth and induced with 0.5 mM IPTG (isopropyl-β-d-thiogalactoside) until the optical density at 600 nm reached 1.0. After a further 16-h incubation at 37 °C, the cells were collected by centrifugation, resuspended in lysis buffer (20 mM Tris-HCl, pH 7.4, 1% Triton X-100 and 20 mM ethylenediaminetetraacetic acid) and lyzed by sonication. Inclusion bodies were recovered by centrifugation at 15 000 *g* for 10 min and were then washed three times with lysis buffer, followed by washing without Triton X-100. The inclusion bodies were dissolved in 20 mM Tris, pH 7.4, containing 6 m GuHCl, 0.5 mM ethylenediaminetetraacetic acid and 10 mM dithiothreitol. The solubilized fraction was clarified by centrifugation and dialyzed against 10 mM HCl to remove dithiothreitol. After dialysis, the sample was redissolved in 6 M GuHCl and added drop-wise into a refolding buffer consisting of 1 m Arg hydrochloride, 0.1 m Tris, pH 8.0, 2 mM ethylenediaminetetraacetic acid, 0.25 mM oxidized glutathione and 0.25 mM reduced glutathione. After incubation at 4 °C overnight, the complex was dialyzed against 10 mM Tris, pH 8.0 and purified to homogeneity using a HisTrap Ni-Sepharose column, HiTrap SP ion-exchange column and Superdex 75 (GE Healthcare). Other PD-L1 variants, such as I54A, Y56A, E58A, D61A, N63A, Q66A, V68A, R113A, M115A, S117A, Y123A and R125A, were prepared using the same procedure. For the preparation of the PD-L1/KN035 complex, the *N*-terminal IgV domain of PD-L1 (amino acids 19–132) was similarly cloned into pET-28a and expressed in *E. coli* as PD-L1. Refolding was performed in refolding buffer containing 0.1 mg ml^−1^ of KN035. The PD-L1 IgV domain/KN035 complexes were subsequently purified using ion-exchange and gel filtration columns (GE Healthcare).

### Crystallization of PD-L1 and its complex with KN035

Both purified PD-L1 and PD-L1/KN035 complexes were concentrated to ~15 mg ml^−1^ and screened for crystallization conditions using commercially available buffer with sitting-drop vapor diffusion; 0.2 μl of the protein complex solution was mixed with 0.2 μl of reservoir solution. Diffraction-quality crystals of PD-L1-IgV/KN035 were obtained at room temperature from 1.4 m ammonium sulfate and 2 m sodium chloride after optimization. The crystals of PD-L1 were grown with a precipitation solution of 0.2 mM ammonium acetate and 20% PEG 3350.

### Structure determination and refinement

Crystals were cryoprotected in 20% glycerol in the mother liquor and flash-cooled in liquid nitrogen. Diffraction data were collected on beamlines BL17U1 (SSRF) and BL19U1 (NCPSS), Shanghai, China [49]. The data were indexed and processed with iMosflm and scaled with Aimless from the CCP4 suite [50]. The initial phases were obtained by molecular replacement using Phaser [51], with PD-L1 and nanobody models derived from PDB entries 1MEL and 4Z18, respectively. The models were subsequently built using Coot [52] and refined using Refmac [53]. Figures were produced with PyMOL software [54]. The atomic coordinates and the structure factors have been deposited in the Protein Data Bank, under accession code PDB 5JDR and 5JDS, respectively.

### Dissociation rate constant

A fortéBio Octet K2 instrument was used to measure the binding kinetics of PD-L1 variants to KN035-Fc with protein A sensor [55]. All sensors were activated in phosphate-buffered saline with 0.1% w/v bovine serum albumin by agitating 96-well microtiter plates at 1 000 r.p.m. to minimize nonspecific interactions. The final volume for all solutions was 200 μl per well. Probes were saturated with 10 μg ml^–1^ KN035 for 40 s before equilibration for 60 s in phosphate-buffered saline with 1% bovine serum albumin. Variants of PD-L1 were prepared as a twofold serial dilution in 0.1% bovine serum albumin and separately incubated with the KN035 bound on the tips for 120 s. Then, the PD-L1 variants were allowed to dissociate for up to 320 seconds depending on the observed dissociation rate. All measurements were corrected for baseline drift by subtracting a control sensor exposed to running buffer only. Data analysis and curve fitting were carried out using Octet software. The affinity of these PD-L1 variants toward PD-1 could not be accurately measured because the affinity between PD-1 and PD-L1 is very low (~6–8 μM) [39, 40], and the affinity of these mutants was even lower. As KN035-Fc is bivalent, we also measured the PD-L1 binding affinity by immobilizing KN035 and KN035-Fc on streptavidin chip surfaces through biotin-labeling, which gave *K*d values of 2.1 nM and 0.84 nM, respectively (data not shown). These *K*d values are largely consistent with the Kd (3.0 nM) derived from KN035-Fc fixed on protein A chip, with differences likely arising from avidity and protein-immobilizing methods.

## Figures and Tables

**Table 1:** Crystallographic data collection and refinement statistics

	*PD-L1*	*PD-L1/KN035 complex*
*Data collection*		
Beamline	SSRF BL17U1	NCPSS BL19U1
Space group	C2 2 21	P61
Cell dimensions		
a, b, c (Å)	72.24, 91.51, 141.83	83.13, 83.13, 73.23
α, β, γ (°)	90, 90, 90	90, 90, 120
Wavelength (Å)	0.9792	0.9785
Resolution (Å)	56.70–2.70 (2.83–2.70)	51.34–1.70 (1.73–1.70)
Total no. of observations	89 216 (12 036)	219 698 (6 935)
Total no. unique	13 282 (1 731)	31 640 (1 657)
*R*_merge_ (%)	12.8 (74.5)	10.4 (88.3)
I σI^−1^	10.8 (2.5)	9.6 (1.5)
Completeness (%)	99.9 (100.0)	99.9 (99.1)
Multiplicity	6.7 (7.0)	6.9 (4.2)
		
*Refinement*		
Resolution (Å)	70.92–2.70 (2.77–2.70)	41.6–1.70 (1.74–1.70)
No. of reflections	12 575 (934)	29 996 (2 193)
No. of residues	418	248
No. of atoms		
Protein	3373	1 888
H_2_O	7	159
Ligand	0	10
*R*_work_/*R*_free_	0.228/0.276	0.178/0.204
B-factors (Å^2^)	51	25
RMSD		
Bond lengths (Å)	0.007	0.011
Bond angles (°)	1.188	1.455
Ramachandran plot		
In preferred region (%)	93.67	97.45
In allowed region (%)	6.08	2.13
Outliers (%)	0.24	0.43

Abbreviations: PD1, programmed death protein 1; RMSDs, root-mean-square deviations.
