# Supplementary material for: Structural basis of a novel PD-L1 nanobody for immune checkpoint blockade
Source: Cell Discov. 2017 Mar 7;3:17004–. doi: 10.1038/celldisc.2017.4 (PMC5341541; doi:10.1038/celldisc.2017.4)
Supplement: Supplementary Information [file celldisc20174-s1.pdf]

Structural basis of a novel PD-L1 nanobody for immune checkpoint blockade

Supplementary Materials

Supplementary methods

Table S1-S2

Figure S1-S7

Supplementary Methods

Competitive binding of KN035 and PD-1 on PD-L1 assessed by ELISA

ELISA plates were coated with PD-L1-Fc at 2μg/ml in 50mM Na_2CO_3/$NaHCO_3$, pH 9.6[47]. After the plates were washed three times with PBST containing 0.05% Tween-20 and blocked with 3% BSA in PBS for 1 hour, serially diluted KN035 were applied to the ELISA plate containing PD-1-hIgG-biotin (10μg/ml) and incubated for 2 hours at 37 °C. Binding was detected with the horseradish peroxidase (HRP)-conjugated goat anti-human IgG, which was developed using tetramethylbenzidine (TMB) substrate and stopped by H_2SO_4. The concentration was determined by absorbance at 450 nm.

Displacement of PD-1 from PD-1/ PD-L1 complex

ELISA plates were coated with PD-L1-Fc at 5μg/ml in 50mM Na_2CO_3/$NaHCO_3$, pH 9.6[47]. After the plates were washed three times with PBST containing 0.05% Tween-20 and blocked with 1% BSA in PBS for 1 hour, PD1-muFc (1 μg/mL) was added and incubated for 2 hours at room temperature. Then serially diluted KN035-Fc/Durvalumab were applied to ELISA plate, incubated for 2 hours at 37 ℃. Binding was assessed by the horseradish peroxidase (HRP)-conjugated goat anti-mouse IgG1 secondary antibody, which was developed using tetramethylbenzidine (TMB) substrate and stopped by H_2SO_4. The concentration was determined by absorbance at 450 nm.

Flow cytometry analysis

Binding property of KN035-Fc with other B7/CD28 superfamily proteins were evaluated by flow cytometry analysis. HEK293T cells were transfected with 2 μg plasmids DNA of hPD-L1-EGFP, hPD-L2-EGFP, hB7H4-EGFP, hCD28-EGFP, hB7H3-EGFP, hICOS-EGFP and mouse PD-L1-EGFP respectively. After 48 hours incubation, the transfected HEK293T cells were suspended and 1×10^6 cells of each sample were stained in 1% BSA/PBS buffer containing 5μg/ml KN035-Fc or mouse anti-ICOS antibody or mouse anti-PD-L2 antibody on ice for 30 mins. After washing, the cells were incubated with secondary antibodyallophycocyanin (APC) conjugated secondary antibody (Biolegend) for 30 mins. The cells were then washed and fixed in 2% formaldehyde/PBS and acquired on GUAVA easy Cyte flowcytometry. Data were analyzed with the GUAVA 5.3.1 program.

Pharmacokinetics of KN035-Fc in Sprague Dawley (SD) rats

In a single-dose pharmacokinetic (PK) study[47], Sprague Dawley rats (5 males and 5 females) received intraperitoneal injection of KN035-Fc at 10 mg/kg dosage. The optical densities (OD) of a set of KN035-Fc concentration standards were determined to plot an OD versus concentration standard curve. KN035-Fc serum concentrations were determined from the standard curve using SoftMax pro software and the data was analyzed by four-parameter curve fitting[47].

Supplementary Tables

Supplementary Table S1. PD-L1 mutants and binding affinities with KN035-Fc

PD-L1 mutation	K_d (M)	$K_{d,mutant}/K_{d,WT}$	mPD-L1 mutation Wang et al.[41]	#Binding to mPD-1 by ELISA[41], %
WT	3.0E-09	1	WT	100
I54A	2.42E-07	80.7	-	-
Y56A	1.24E-06	413.3	Y56S	100
E58A	1.49E-07	49.7	E58S	300
D61A	1.99E-08	6.6	-	-
N63A	2.30E-08	7.7	-	-
Q66A	4.88E-07	162.7	-	-
V68A	2.76E-08	9.2	-	-
R113A	5.34E-07	178	C113Y	300
M115A	5.51E-08	18.4	I115A	3
S117A	1.26E-08	4.2	S117Y	100
Y123A	4.24E-08	14.1	-	-
R125A	2.97E-08	9.9	-	-

#Binding of mPD-L1 variants to mPD-1 was assessed by performing an ELISA in Wang *et al.*[41].

Supplementary Table S2. Polar interactions between KN035 and PD-L1 (distance ≤ 3.5 Å)

Hydrogen bonds and salt bridges			
KN035 contact residue	KN035 residue location	PD-L1 contact residue	PD-L1 residue location
S30	CDR1	D61	CC’ loop
S35	CDR1	D61	CC’ loop
R37	CDR1	D61	CC’ loop
D107	CDR3	R113	F strand
S108	CDR3	E58	C strand
E110	CDR3	Y56	C strand
D111	CDR3	Q66	C’ strand
T111.2	CDR3	Q66	C’ strand
Q116	CDR3	R125	G strand
Water-mediated hydrogen bonds			
S108	CDR3	D61	CC’ loop
T112.2	CDR3	S117	F strand
S112.1	CDR3	A121	G strand
G113	CDR3	A121, D122	G strand

Supplementary Figures

A

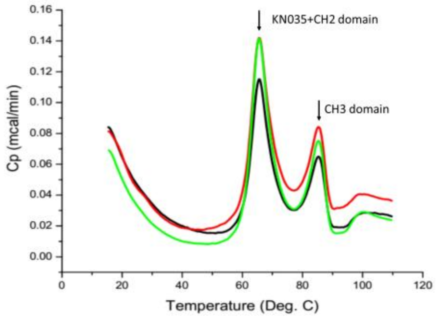

B

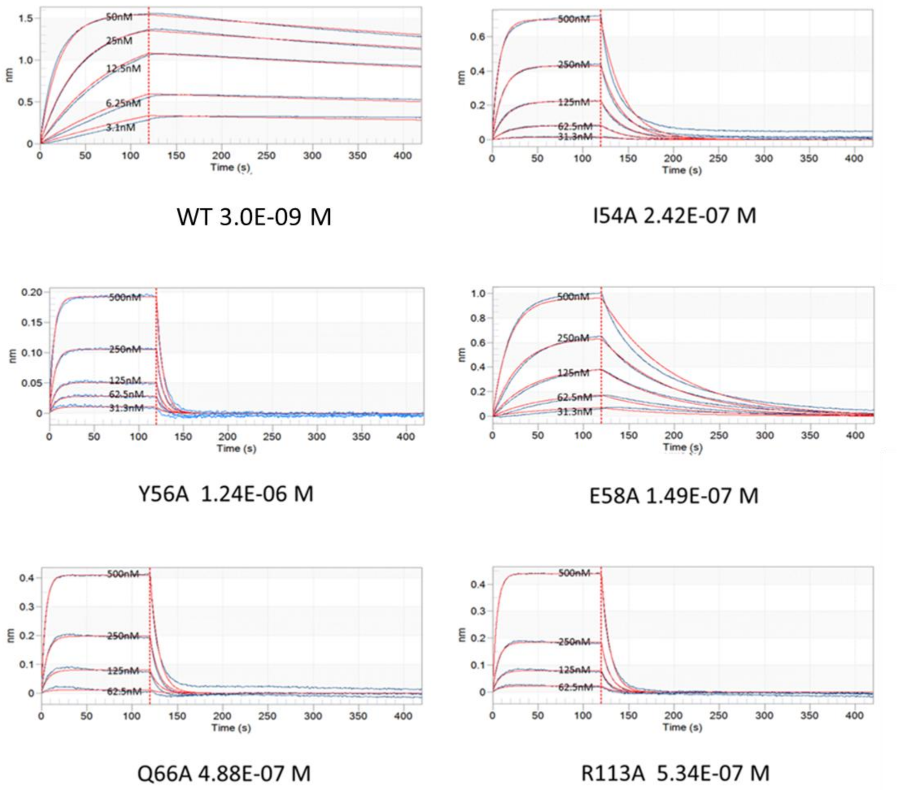

Figure S1. A. The thermal stability of KN035-Fc. The thermal stability of three preparations of KN035-Fc in PBS was measured by differential scanning calorimetry (DSC) on a MicroCal VP-Capillary DSC (GE Healthcare). The proteins were heated from 10 ℃ to 110 ℃ at a rate of 110 ℃/hour. The thermogram was fitted using the Cp value versus temperature after subtracting the buffer reference scan, with the first transition of the CH2+KN035 domain unfolding (Tm ≈ 66 ℃) and the second transition of the CH3 domain unfolding (Tm ≈ 85 ℃). B. Binding affinities of KN035-Fc with PD-L1 mutants. Serial dilutions of the PD-L1 mutants were applied to KN035-Fc immobilized on an Fc sensor chip. The blue lines represent the measured data, and the red lines show the fitted curves.

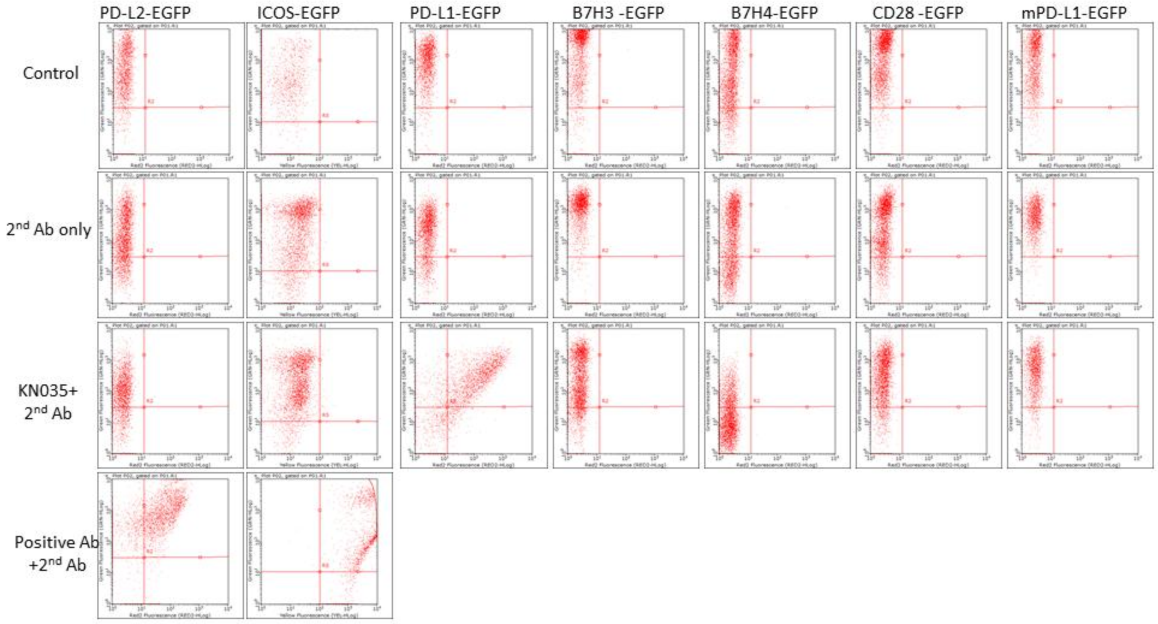

Figure S2. Binding specificity of KN035-Fc to various members of the B7/CD28 superfamily. HEK293T cells were transfected with PD-L1-EGFP, PD-L2-EGFP, mPD-L1-EGFP, B7H4-EGFP, B7H3-EGFP, and ICOS-EGFP and then incubated with KN035 or positive antibodies (anti-PD-L2 and anti-ICOS). The cells were stained with a secondary antibody and were detected by flow cytometry. The red fluorescence from allophycocyanin (APC)-labeled secondary antibody is shown on the abscissa, and the green fluorescence from the EGFP fusion protein is shown on the ordinate. The results showed that KN035-Fc could specifically bind PD-L1. The positive antibody recognized the corresponding ligands PD-L2 and ICOS.

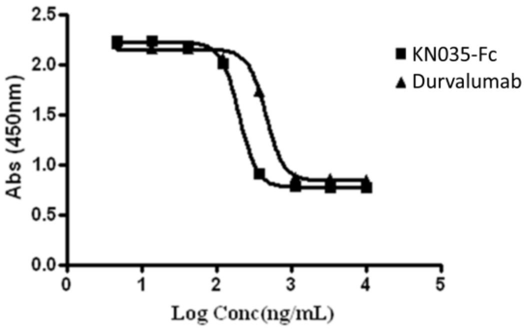

Figure S3. Displacement of PD-1 from the PD-1/PD-L1 complex by KN035-Fc and Durvalumab. Plates coated with human PD-L1 were incubated with PD1-muFc (1 μg/mL) for 2 hours at RT. Serially diluted KN035-Fc/Durvalumab antibodies were applied to the ELISA plate and incubated for 2 hours at 37 ℃. Binding was detected with a horseradish peroxidase (HRP)-conjugated goat anti-mouse IgG1 secondary antibody. The EC_{50} for KN035-Fc and Durvalumab to displace PD-1 is 198.3 ng/mL (2.5 nM) and 452.7 ng/ml (3.1 nM), respectively.

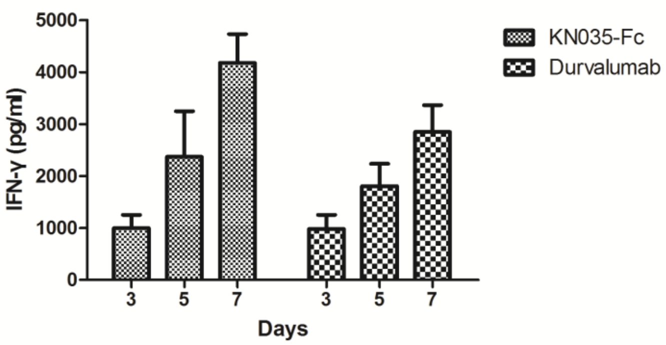

Figure S4. The time course of IFN-γ secretion by CD4+ cells after incubation with KN035-Fc and Durvalumab. IFN-γ levels were determined on day 3, 5, and 7 after the incubation with the antibodies KN035-Fc and Durvalumab (2.51 nM).

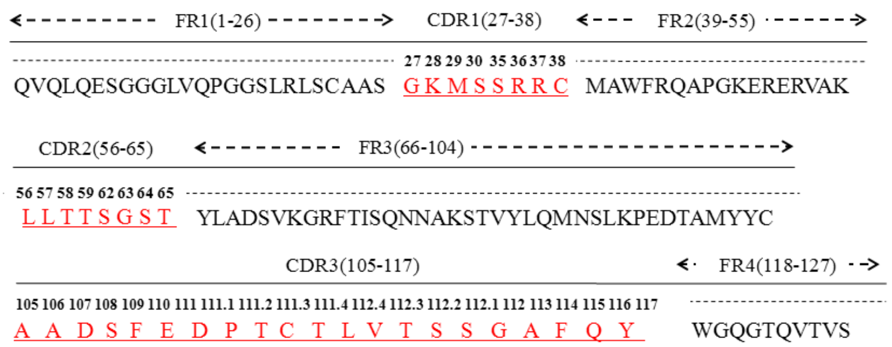

Figure S5. Amino acid sequence of KN035, numbered according to the IMGT numbering[29].

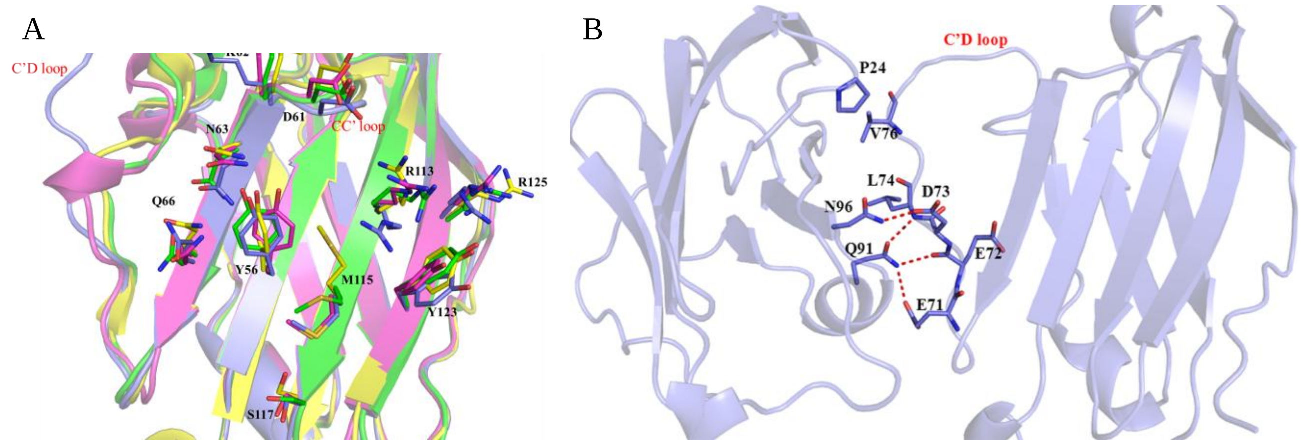

Figure S6. Conformational changes of PD-L1. (A) Overlaid structures of the IgV domains of PD-L1. The structures of the PD-L1 IgV domains from the PD-1/PD-L1 complex (PDB: 4ZQK, magenta), the KN035/PD-L1 complex (slate), the free PD-L1 structure obtained here (green) and the previously reported free PD-L1 structure (PDB: 5C3T, yellow) are superimposed in this cartoon. The C'D loop of PD-L1 shifts approximately 7.5 Å in the PD-L1/KN035 complex, which is likely induced by crystal packing (B). The other minor conformational changes of the mainchain amongst the PD-L1 molecules, especially the changes between the two free PD-L1 structures (green and yellow), likely reflect the thermal dynamics of the PD-L1 molecule. The conformations of the key residues shown as sticks indicate the flexibility of these sidechains in the structures of the free form of PD-L1 (green and yellow), as they adopt slightly altered conformations following PD-1 or KN035 binding. This result is consistent with previous NMR studies of PD-1/PD-L1 interactions that show movements in the surface residues during binding[40]. (B) The movement of the C'D loop of PD-L1 following KN035 binding shown in A is induced by crystal packing. Residues 71-74 and 76 from one PD-L1 molecule form hydrogen bonds or hydrophobic interactions with other residues, including 24, 91, and 96, from a neighboring PD-L1 molecule. The residues at the interface are shown as slate sticks. Dashed lines represent polar interactions.

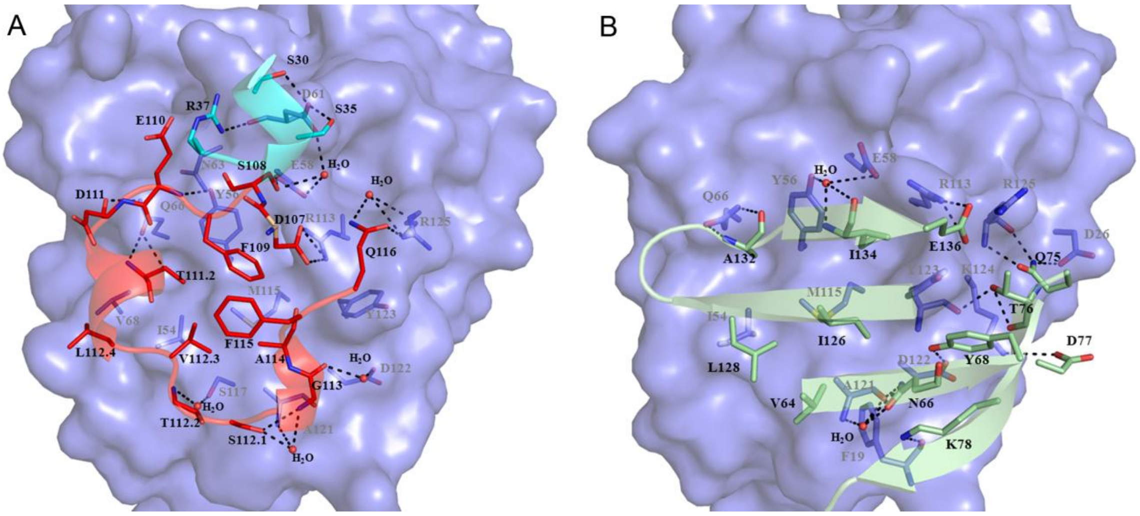

Figure S7. The detailed binding interactions at the interfaces of KN035/PD-L1 (A) and PD-1/PD-L1 (B). Residues of KN035 in the binding interface are shown as red or cyan sticks and, those of PD-1 are shown as green sticks. Hydrogen bonds are depicted as black dashed lines, and water molecules are depicted as red spheres.
